# Metallothionein 1G functions as a tumor suppressor in thyroid cancer through modulating the PI3K/Akt signaling pathway

**DOI:** 10.1186/1471-2407-13-462

**Published:** 2013-10-08

**Authors:** Jiao Fu, Hongjun Lv, Haixia Guan, Xiaoying Ma, Meiju Ji, Nongyue He, Bingyin Shi, Peng Hou

**Affiliations:** 1 Department of Endocrinology, The First Affiliated Hospital of Xi’an Jiaotong University School of Medicine, Xi’an 710061, China; 2 Department of Endocrinology and Metabolism, The First Affiliated Hospital of China Medical University, Shenyang 110001, China; 3 Center for Translational Medicine, The First Affiliated Hospital of Xi’an Jiaotong University School of Medicine, Xi’an 710061, China; 4 State Key Laboratory of Bioelectronics, Southeast University, Nanjing 210096, China

**Keywords:** Thyroid cancer, Metallothionein 1G (MT1G), DNA methylation, PI3K/Akt pathway, Rb/E2F pathway

## Abstract

**Background:**

*MT1G* inactivation mediated by promoter methylation has been reported in thyroid cancer. However, the role of *MT1G* in thyroid carcinogenesis remains unclear. The aim of this study is to examine the biological functions and related molecular mechanisms of *MT1G* in thyroid cancer.

**Methods:**

Methylation-specific PCR (MSP) was performed to analyze promoter methylation of *MT1G* and its relationship with clinicopathological characteristics of papillary thyroid cancer (PTC) patients. Conventional and real-time quantitative RT-PCR assays were used to evaluate mRNA expression. The functions of ectopic *MT1G* expression were determined by cell proliferation and colony formation, cell cycle and apoptosis, as well as cell migration and invasion assays.

**Results:**

*MT1G* expression was frequently silenced or down-regulated in thyroid cancer cell lines, and was also significantly decreased in primary thyroid cancer tissues compared with non-malignant thyroid tissues. Promoter methylation, along with histone modification, contributes to *MT1G* inactivation in thyroid tumorigenesis. Moreover, our data showed that *MT1G* hypermethylation was significantly positively associated with lymph node metastasis in PTC patients. Importantly, restoring *MT1G* expression in thyroid cancer cells dramatically suppressed cell growth and invasiveness, and induced cell cycle arrest and apoptosis through inhibiting phosphorylation of Akt and Rb.

**Conclusions:**

We have for the first time revealed that *MT1G* appears to be functional tumor suppressor involved in thyroid carcinogenesis mainly through modulating the phosphatidylinositol-3-kinase (PI3K)/Akt pathway and partially through regulating the activity of Rb/E2F pathway in this study.

## Background

Thyroid cancer is the most common malignant tumor in endocrine system, and its incidence has been steadily increasing in many regions of the world [1,2]. Follicular epithelial cell-derived thyroid tumors are the most common type, accounting for about 95-97% of all thyroid malignancies, and are histologically classified into follicular adenoma (FA), papillary thyroid cancer (PTC), follicular thyroid cancer (FTC), and anaplastic thyroid cancer (ATC). PTC and FTC are differentiated thyroid cancer as they possess differentiated features of their origin cells and have a good prognosis. ATC is an ultimate undifferentiated thyroid cancer with an inexorable fatal outcome and generally fails to respond to available chemo- and radiotherapy. Poorly differentiated thyroid cancers (PDTCs) are those within intermediate histopathological patterns between differentiated and undifferentiated thyroid cancers [3,4].

Like other cancers, thyroid carcinogenesis involves gradual accumulation of various genetic and epigenetic alterations, leading to gain-of-function in oncogenes and loss-of-function in tumor suppressor genes [5,6]. Expanded knowledge of genetic events occurring in thyroid cancer has improved our understanding of thyroid tumorigenesis and provided new insights into thyroid cancer management. Most of these events are closely bound up with aberrant signaling of MAPK and phosphatidylinositol-3-kinase (PI3K)/Akt pathways, which are crucial for tumor initiation and progression. For example, rearrangement of *RET*/*PTC* and mutations of *BRAF* and *RAS* account for approximately 70% of overactivation of MAPK signaling, leading to PTC initiation, while the alterations affecting PI3K/Akt pathway, such as mutations of *RAS*, *PTEN* and *PIK3CA*, amplification of *PIK3CA* and rearrangement of *PAX8*/*PPARγ*, are extensive in FTC. Despite of the initiating role in FTC, the coexistence of PI3K/Akt pathway-related genetic alterations is also found to play a role in facilitating progression and dedifferentiation in thyroid cancer [5,7,8].

In addition to genetic factors, epigenetic events, such as aberrant promoter methylation, play a key role in human carcinogenesis [9], including thyroid cancer [6,10]. Promoter methylation is one of the major mechanisms to inactivate tumor-related genes, particularly tumor suppressor genes, along with genetic events, ultimately leading to carcinogenesis [9,11]. Significantly, promoter methylation is now regarded as an important hallmark of cancer cells, and plays a significant role in tumor transformation and progression, impacting the clinical outcome of cancer patients [12,13].

Metallothionein 1G (*MT1G*), a member of Metallothioneins (MTs), is a highly conserved, low-molecular weight (6–7 kDa), and cysteine residues-rich protein [14,15]. Most of the biological functions proposed for MTs are related to metal-binding property, including detoxification of heavy metals, donation of zinc/copper to certain enzymes and transcription factors and protection against oxidative stress [16-18]. Previous studies showed that *MT1G* expression was repressed by promoter methylation in several human cancers, including hepatocellular cancer, colorectal cancer, prostate cancer and thyroid cancer [19-22]. Moreover, restoration of *MT1G* expression in thyroid cancer cells inhibited cell growth i*n vitro* and *in vivo*, suggesting an oncosuppressor role [23]. However, the molecular mechanisms underlying *MT1G* as a tumor suppressor in thyroid cancer remain totally unknown. In the present study, our data indicated that *MT1G* hypermethylation was frequently found in PTC and significantly associated with lymph node metastasis. Importantly, our data for the first time revealed that ectopic expression of *MT1G* in thyroid cancer cells dramatically inhibited cell growth and invasiveness, and induced cell cycle arrest and apoptosis via modulating the activity of PI3K/Akt pathway.

## Methods

### Clinical samples and DNA isolation

With the institution review board approval, a total of 244 paraffin-embedded thyroid tissues were randomly obtained from the First Affiliated Hospital of Xi’an Jiaotong University School of Medicine (Xi’an, P.R. China), including 178 PTCs, 16 FTCs, 9 medullary thyroid cancers (MTCs), 9 ATCs, and 32 goiters. None of these patients received chemotherapy or radiotherapy before the surgery. Informed consent was obtained from each patient before the surgery. All of the samples were histologically examined by a senior pathologist at Department of Pathology of the Hospital to identify the clinicopathological characteristics of the tumors, which were presented in Table 1. The genomic DNA was isolated from paraffin-embedded tissues as previously described [7], using xylene to remove the paraffin and sodium dodecyl sulfate (SDS) and proteinase K to digest tissues, followed by standard phenol-chloroform extraction and ethanol precipitation of DNA. Extraction of total RNA from paraffin-embedded tissues was performed using E.Z.N.A. FFPE RNA Kit (Omega Bio-Tek Inc., GA) according to manufacturers’ instruction.

**Table 1:** Clinical profile of thyroid cancer patients and controls

**Characteristics**	** No. of patients (%)**				
	**PTC (n=178)**	**FTC (n=16)**	**MTC (n=9)**	**ATC (n=9)**	**Goiter (n=32)**
Gender					
Male	48 (27.0)	3 (18.8)	4 (44.4)	4 (44.4)	2 (6.3)
Female	130 (73.0)	13 (81.3)	5 (55.6)	5 (55.6)	30 (93.8)
Age (years, mean ± SD)	42.1 ± 15.3	49.5 ± 14.5	53.6 ± 9.5	65.6 ± 9.7	48.7 ±15.0
≤30	41 (23.0)	1 (6.3)	0 (0.0)	0 (0.0)	6 (18.8)
30-50	92 (51.7)	7 (43.8)	4 (44.4)	1 (11.1)	9 (28.1)
50-70	33 (18.5)	7 (43.8)	5 (55.6)	5 (55.6)	15 (46.9)
>70	12 (6.7)	1 (6.3)	0 (0.0)	3 (33.3)	2 (6.3)
Tumor size (cm^3^)*					
≤1	19 (14.8)	3 (42.9)	0 (0.0)	0 (0.0)	
1-3	34 (26.6)	1 (14.3)	0 (0.0)	1 (33.3)	
3-5	25 (19.5)	0 (0.0)	0 (0.0)	0 (0.0)	
>5	50 (39.1)	3 (42.9)	1 (11.1)	2 (66.7)	
Tumor stage					
I	109 (61.2)	6 (37.5)	1 (11.1)	0 (0.0)	
II	25 (14.0)	3 (18.8)	2 (22.2)	0 (0.0)	
III	43 (24.2)	7 (43.8)	6 (66.7)	0 (0.0)	
IV	1 (0.6)	0 (0.0)	0 (0.0)	9 (100.0)	
Invasion					
No	98 (55.1)	11 (68.8)	6 (66.7)	3 (33.3)	
Yes	80 (44.9)	5 (31.3)	3 (33.3)	6 (66.7)	
Lymph node metastasis					
No	92 (51.7)	13 (81.3)	2 (22.2)	6 (66.7)	
Yes	86 (48.3)	3 (18.7)	7 (77.8)	3 (33.3)	
Recurrence					
No	158 (88.8)	11 (68.8)	7 (77.8)	6 (88.9)	
Yes	20 (11.2)	5 (31.3)	2 (22.2)	3 (11.1)	

*Only 140 patients have the information of tumor size.

### Cell culture

Human thyroid cancer cell lines BCPAP, FTC133, IHH4, K1, 8305C and the normal thyroid epithelial cell-derived cell line HTori-3 were from Dr. Haixia Guan (The First Affiliated Hospital of China Medical University, Shenyang, P.R. China). C643 was from Dr. Lei Ye (Ruijin Hospital, Shanghai, P.R. China). The origins and genetic alterations of these thyroid cancer cells were summarized in (see Additional file 1: Table S1). These cells were all routinely cultured at 37°C in RPMI 1640 medium with 10% fetal bovine serum (FBS), except for FTC133 that was cultured in DMEM/Ham’s F-12 medium (Invitrogen Technologies, Inc., CA). All media were supplemented with penicillin/streptomycin. For some experiments, cells were treated with DNA methyltransferase (DNMT) inhibitor 5-aza-2′-deoxycytidine (5-Aza-dC) or/and histone deacetylase (HDAC) inhibitor suberoylanilide hydroxamic acid (SAHA) as the indicated concentrations and time, and medium and agents were replenished every 24 h. The powder of 5-Aza-dC and SAHA were obtained from Sigma-Aldrich and Cayman Chemical, and dissolved in 50% acetic acid/50% PBS and DMSO, respectively. The same volumes of the vehicle (50% acetic acid/50% PBS or DMSO) were used as the controls.

### RNA extraction, conventional RT-PCR and real-time quantitative RT-PCR

Total RNA was extracted using TRIzol reagent (Takara Inc., Dalian, P.R. China) according to the instructions of manufacturer. one μg of total RNA was converted to cDNA using PrimeScript RT reagent Kit (Takara Inc., Dalian, P.R. China) according to the instructions of the manufacturer. Conventional RT-PCR was carried out to amplify *MT1G*. The *β*-*actin* gene was run in parallel for quality. PCR products were resolved by 1.5% agarose gel electrophoresis and visualized by ethidium bromide staining. Real-time quantitative PCR assay was performed to evaluate the expression of *MT1G*, *E*-*cadherin*, *Vimentin*, *Snail*, *Slug*, and *Twist* on a CFX96 Thermal Cycler Dice™ real-time PCR system (Bio-Rad Laboratories, Inc., CA), using SYBR Premix Ex*Taq* II (Takara Inc., Dalian, P.R. China) according to the instructions of manufacturer. The expression value of each gene was normalized to *18S* rRNA cDNA to calculate the relative amount of RNA present in each sample according to the2^-ΔΔCt^ method [24]. Each sample was run in triplicate. The primer sequences were presented in (see Additional file 1: Table S2).

### Sodium bisulfite treatment and methylation-specific PCR (MSP)

Genomic DNA was treated with sodium bisulfite as described previously [25]. Briefly, a final volume of 20 μL of H_2_O containing 2 μg genomic DNA, 10 μg salmon sperm DNA, and 0.3M NaOH was incubated at 50°C for 20 min to denature the DNA. The mixture was then incubated for 2 h at 70°C in 500 μL of a freshly prepared solution containing 3 M sodium bisulfite (Sigma, Saint Louis, MO) and 10 mM hydroquinone (Sigma, Saint Louis, MO). DNA was subsequently purified with a Wizard DNA Clean-Up System (Promega Corp., Madison, WI) following the instructions of the manufacturer, followed by ethanol precipitation, dry, and resuspension in 50 μL of deionized H_2_O. Bisulfited-treated DNA samples were stored at −80°C until use.

MSP was performed in a final reaction mixture of 20 μL containing 50 ng of bisulfite-treated DNA, 16.6 mM of ammonium sulfate, 67 mM of Tris (pH 8.8), 2 mM MgCl_2_, 200 μM each of deoxynucleotide triphosphate mixture (dATP, dCTP, dGTP, and dTTP), 200 nM forward and reverse primers, and 0.5 U of platinum *Taq* DNA polymerase (Invitrogen Technologies, Inc., CA). The PCR was run in a Thermal cycler (Bio-Rad Laboratories, Inc., CA) as follows: after a 4-min denaturation at 95°C, the reaction was run 35 cycles, each comprising 45 s of denaturing at 95°C, 45 s of annealing at variable temperatures according to the primers, and 45 s of extension at 72°C, with an extension at 72°C for 5 min as the last step. Normal leukocyte DNA was methylated *in vitro* with Sss I methylase (New England Biolabs, Beverly, MA) to generate completely methylated DNA as a positive control. Methylation-specific primers were: 5′- TCG TAT ACG GGG GGT ATA GC-3′ (forward) and 5′- GCG ATC CCG ACC TAA ACT -3′ (reverse), and Unmethylation-specific primers were: 5′- AAGTTGTATATGGGGGGTATAGT-3′ (forward) and 5′- CCCACAATCCCAACCTAAACT -3′(reverse). The PCR products were electrophoresed on a 1.2 % agarose gel and visualized under UV illumination.

### Plasmid constructs and transfection

The full-length *MT1G* open reading frame was amplified from human thyroid epithelial cell line HTori-3 by RT-PCR, and cloned into mammalian expression vector pEGFP-N1. Thyroid cancer cells were transfected with pEGFP-N1-*MT1G* or pEGFP-N1 (empty vector) using X-tremeGene HP DNA Transfection Reagent (Roche Applied Science, Germany) according to the manufacturer’s protocol. After 48 h of transfection, the transfectants were selected in a medium containing 0.5 mg/mL of G418 for 2 to 3 weeks to generate the stable pools.

### Western blot analysis

Cells were lysed in RIPA buffer. Cellular proteins were collected and subjected to 10% SDS-PAGE, and transferred onto PVDF membranes (Amersham Pharmacia Biotech, Piscataway, NJ). The membranes were then incubated with specific primary antibodies. Anti-phospho-Akt^Ser473^, anti-phospho-Akt^Thr308^, anti-total-Akt (t-Akt), and anti-phospho-Erk1/2 were purchased from Bioworld Technology, co, Ltd. Anti-p53 and anti-Mdm2 were purchased from Santa Cruz Biotechnology, Inc. Anti-E-cadherin, anti-Vimentin, anti-phospho-Rb^Ser811^ and anti-Rb were purchased from Epitomics, Inc. Anti-Bak and anti-GAPDH were purchased from Abgent, Inc. Anti-phospho-p70S6K was purchased from R&D Systems, Inc. Anti-p21 was purchased from Cell Signaling Technology, Inc. Anti-Smac was purchased from Abcam. This was followed by incubation with horseradish peroxidase-conjugated anti-rabbit or anti-mouse IgG antibodies from Santa Cruz Biotechnology, Inc., and antigen-antibody complexes were visualized using the Western Bright ECL detection system (Advansta, CA).

### Cell proliferation and colony formation assays

Cells stably transfected with pEGFP-N1-*MT1G* or empty vector were plated in 96-well plates and cultured with 0.5% FBS. MTT assay was performed daily over a 4-d time course to evaluate cell proliferation. Cell culture was added with 10 μL of 5 mg/mL MTT agent (Sigma, Saint Louis, MO) and incubated for 4 h, followed by addition of 150 μL of DMSO and further 15-min incubation. The plates were then read on a microplate reader using a test wavelength of 570 nm and a reference wavelength of 670 nm. Three triplicates were done to determine each data point.

For colony formation assay, cells (5 × 10^5^cells per well) were seeded in 6-well plates and transfected with pEGFP-N1-*MT1G* or empty vector. After 48 h, the transfectants were replated in 12-well plate at a density of 300 cells per well and subjected to G418 (500 μg/mL) for 14 days. The selective medium was refreshed every 3 days. Surviving colonies (≥50 cells per colony) were fixed with methanol, stained with 1.25% crystal violet and counted under a light microscope. The experiments were similarly performed in triplicate.

### Cell cycle and apoptosis assays

For cell cycle analysis, transiently transfected cells were harvested, washed twice in PBS, and fixed in 70% ethanol on ice for at least 30 min. Cells were then stained with propidium iodide solution (50 μg/mL propidium iodide, 50 μg/mL RNase A, 0.1% Triton-X, 0.1mM EDTA). Cell cycles were analyzed based on DNA contents by FACS using a Flow Cytometer (BD Biosciences, NJ).

Apoptosis assays were performed by the use of Hoechst 33342 (Sigma-Aldrich, Saint Louis, MO) staining as previously described [26]. Briefly, transiently transfected cells were stained with 10 μg/mL of Hoechst 33342 at 37°C for 30 min. After PBS washing, the stained cells were imaged with a digital camera attached to a fluorescence microscope (Olympus IX71). For quantitation of the number of apoptotic cells, 500 cells were counted under microscope, and characteristic morphology of apoptotic nuclei was defined as previously described [27]. All the experiments were performed in duplicate.

### Cell migration and invasion assays

Cell migration and invasion assays were performed using Transwell chambers (8.0 μm pore size; Millipore, MA), which were coated with or without Matrigel (4 × dilution; 60 μL/well; BD Bioscience, NJ), in 24-well plates. Chambers were pre-coated with rat tail tendon collagen type 1 (0.5 mg/mL) on the lower surface. Cells stably transfected with pEGFP-N1-*MT1G* or empty vector were starved overnight and then seeded in the upper chamber at a density of 2 × 10^5^cells/mL in 400 μL of medium containing 0.5% FBS. Medium with 10% FBS (600 μL) was added to the lower chamber. Following a 24 h-incubation at 37°C with 5% CO_2_, non-migrating (or non-invading) cells in the upper chamber were removed with a cotton swab, and migrating (or invading) cells were fixed in 100% methanol and stained with 0.5% crystal violet in 2% ethanol. Photographs were taken randomly for at least four fields of each membrane. The number of migrating (or invading) cells was expressed as the average number of cells per microscopic field over four fields.

### Scratch wound-healing assay

Cells were cultured in standard medium until they were 80-90% confluent on the day of transfection. After 48 h of transfection, cells were starved by medium containing 0.5% serum overnight. The wounds were scratched using 200 μl sterile pipette tips. Cells were then cultured in medium containing 1% serum to facilitate cell migration into the wounded area. The widths of wound were measured and photographed under a phase-contrast microscope. Each experiment was performed in triplicate wells for three times.

### Statistical analysis

The SPSS statistical package (16.0, Chicago, IL, USA) was used for data analysis. Independent sample *t* and *χ2* tests were used to analyze continuous and categorical variables, respectively. The risk of *MT1G* hypermethylation to clinicopathological characteristics was analyzed using univariate or multivariate logistic regression. All of the statistical tests were two-sided. A *P* < 0.05 was considered to be statistically significant.

## Results

### Frequent down-regulation and promoter hypermethylation of *MT1G* in primary thyroid cancers

Similar to the findings in a previous study (23), *MT1G* expression was significantly down-regulated in PTC tissues compared with non-malignant tissues (*P* =0.0001) (see Additional file 1: Figure S1A). It has been well documented that aberrant promoter methylation is related to gene silencing. We next analyzed the methylation status of *MT1G* by methylation-specific PCR (MSP). A typical CpG island spans the promoter region of *MT1G*, and the position of MSP primers is indicated in (see Additional file 1: Figure S1B). *MT1G* hypermathylation was found in 30.2% (64/212) of thyroid cancers, including 31.5% (56/178) of PTC, 25.0% (4/16) of FTC, 22.2% (2/9) of MTC, and 22.2% (2/9) of ATC. In addition, it was also found in 18.8% (6/32) of goiter. These data suggested that *MT1G* was more frequently methylated in thyroid cancer tissues compared with non-malignant thyroid tissues (see Additional file 1: Table S3). MSP results of 2 representative PTC samples were shown in (see Additional file 1: Figure S1C).

### Association of *MT1G* hypermethylation with lymph node metastasis in PTC

Because frequent *MT1G* hypermethylation was demonstrated in thyroid cancers, particularly in PTC, the association of *MT1G* hypermethylation with clinicopathological characteristics was analyzed in a total of 178 PTC. As shown in Table 2, we failed to find a significant relationship between *MT1G* hypermethylation and most of clinicopathological characteristics, such as gender, age, tumor invasion, tumor stage, tumor size, and tumor recurrence. However, the univariate analysis revealed that *MT1G* hypermethylation was associated with a significantly increased risk of lymph node metastasis (OR =2.14, 95% CI =1.12-4.07; *P* =0.02). In order to assess the independent association of *MT1G* hypermethylation with gender, age, tumor invasion, lymph node metastasis, tumor stage, and tumor recurrence, we further performed multivariate logistic regression. Similar to univariate analysis, after adjustment, *MT1G* hypermethylation remained significantly positively associated with lymph node metastasis (OR=2.40, 95% CI=1.19-4.83) (see Additional file 1: Table S4), suggesting that *MT1G* hypermethylation might be an independent factor in predicting lymph node metastasis for PTC patients.

**Table 2:** ***MT1G *hypermethylation in PTC** ― **univariate associations with clinicopathological characteristics (OR**^**1 **^**and 95% CI)**

**Variable**	**OR**^**1**^	**95% CI**	***P *value**
Gender	0.99	0.48-2.01	0.97
Age^2^	0.92	0.63-1.36	0.69
Tumor invasion	0.71	0.38-1.36	0.31
Lymph node metastasis	2.14	1.12-4.07	0.02*
Tumor stage^3^	0.97	0.67-1.40	0.87
Tumor size^4^	1.11	0.78-1.58	0.55
Tumor recurrence	1.19	0.47-2.99	0.71

^1^*OR*: odds ratio with 95% confidence interval.

^2^Age (≤30y; 30-50y; 50-70y; >70y).

^3^Tumor stage (I, II, III, IV).

^4^Tumor size (≤1 cm; 1–3 cm; 3–5 cm; >5 cm).

*Significant at *P* <0.05.

### Epigenetic silencing of *MT1G* in thyroid cancer cells

**Figure 1:**
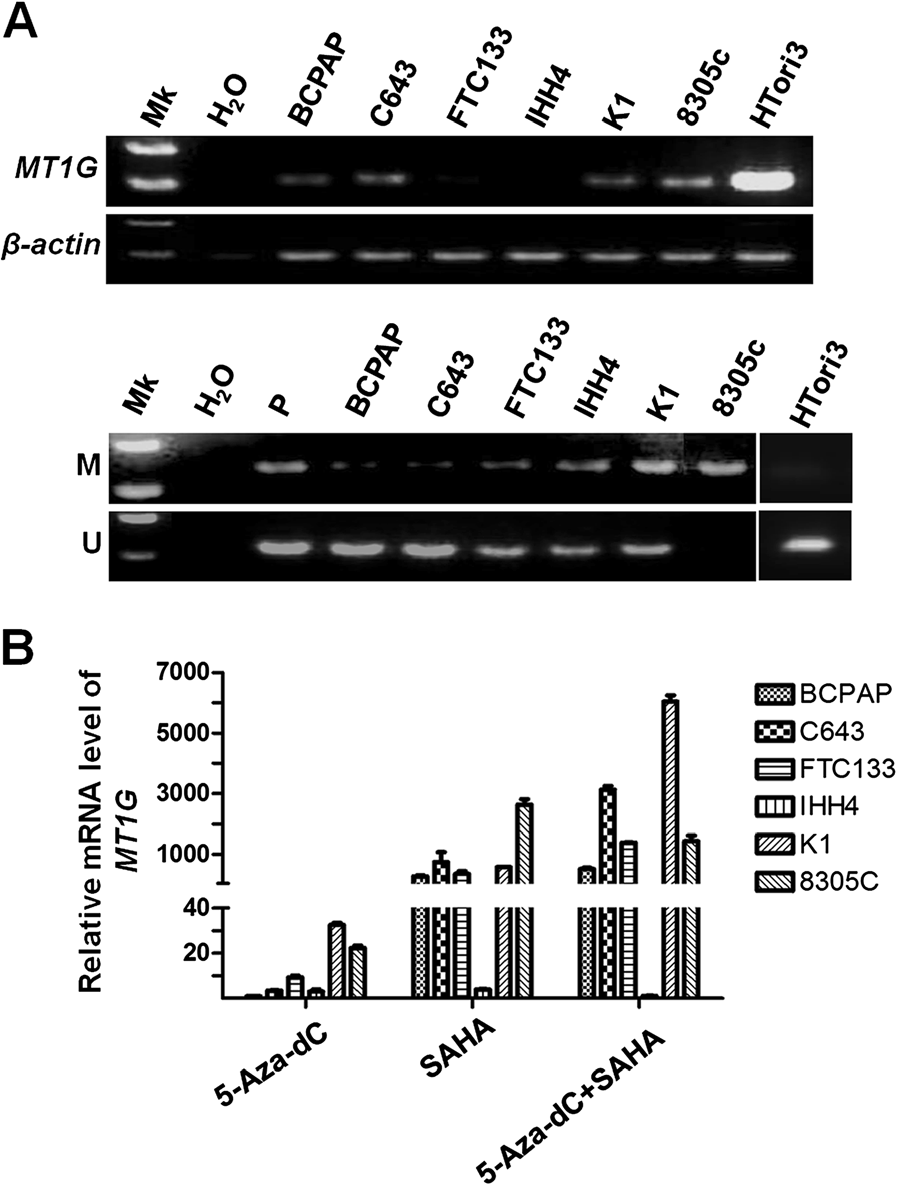
***MT1G *inactivation mediated by epigenetic alterations in thyroid cancer. (A)** Frequent down-regulation and promoter methylation of *MT1G* in thyroid cancer cell lines. *MT1G* expression was determined by semi-quantitative RT-PCR with *β*-actin as an internal control (upper panel). Promoter methylation of *MT1G* in cell lines was determined by MSP (lower panel). Mk, DNA marker; P, positive control for methylated gene. **(B)***MT1G* expression was restored in thyroid cancer cells treated with 5-Aza-dC and SAHA alone or in combination. Real-time quantitative RT-PCR was performed to evaluate mRNA expression of *MT1G*, and *18S* mRNA was used as a normalized control. Data are presented as mean ± SD of values from three different assays.

To determine whether *MT1G* expression is regulated by epigenetic mechanisms in thyroid cancer, such as promoter methylation and histone modification, we examined *MT1G* expression in 6 thyroid cancer cell lines by conventional RT-PCR. As shown in Figure 1A (upper panel), *MT1G* expression was silenced or down-regulated in all thyroid cancer cell lines compared with normal thyroid epithelial cell line HTori3. *MT1G* hypermethylation was also found in these cell lines, particularly 8305c cells that showed complete methylation (Figure 1A, lower panel). However, down-regulation or silencing of *MT1G* was not completely consistent with methylation status of its promoter. For example, methylation level of *MT1G* was not high in FTC133 cells, although its expression was nearly undetected. Thus, we supposed that other epigenetic mechanisms, such as histone modification, along with DNA methylation, were involved in *MT1G* inactivation in thyroid cancer cells. To explore this, thyroid cancer cell lines were treated with a DNMT inhibitor, 5-Aza-dC, and a HDAC inhibitor, SAHA, alone or in combination. *MT1G* expression was then analyzed using real-time quantitative RT-PCR. As shown in Figure 1B, 5-Aza-dC treatment only caused partial reactivation of *MT1G* in most of cancer cell lines. Compared with 5-Aza-dC treatment alone, *MT1G* expression was more significantly restored in these cancer cells treated with SAHA alone or in combination with 5-Aza-dC. Of them, *MT1G* expression was most significantly induced by these inhibitors in K1 cells. These data suggested that epigenetic alterations would be a major mechanism to inactivate *MT1G* in thyroid cancer cells.

### MT1G inhibits thyroid cancer cell growth

Frequent down-regulation or silencing of *MT1G* mediated by epigenetic alterations in thyroid cancer cell lines and primary thyroid cancers but not in non-malignant thyroid tissues implicated that *MT1G* may be a tumor suppressor. To test this speculation, we examined the growth inhibitory effect through ectopic expression of *MT1G* in K1, FTC133, BCPAP and C643 cells, wherein *MT1G* expression was relatively low and could be dramatically induced by 5-Aza-dC and SAHA. *MT1G* re-expression in the transfected cells was confirmed by conventional and real-time quantitative RT-PCR, respectively (see Additional file 1: Figure S2A). Ectopic expression of *MT1G* caused a decrease in cell proliferation, particularly in K1 and FTC133 cells (see Additional file 1: Figure S2B). The inhibitory effect on thyroid cancer cell growth was further confirmed by colony formation assay. As shown in (see Additional file 1: Figure S2C), the colonies formed in *MT1G*-transfected cells were fewer and smaller than those formed in empty vector-transfected cells, particularly in K1 cells. Taken together, MT1G exhibits the growth inhibitory ability in thyroid cancer cells and acts as a potential tumor suppressor.

### MT1G induces cell cycle arrest and apoptosis of thyroid cancer cells

**Figure 2:**
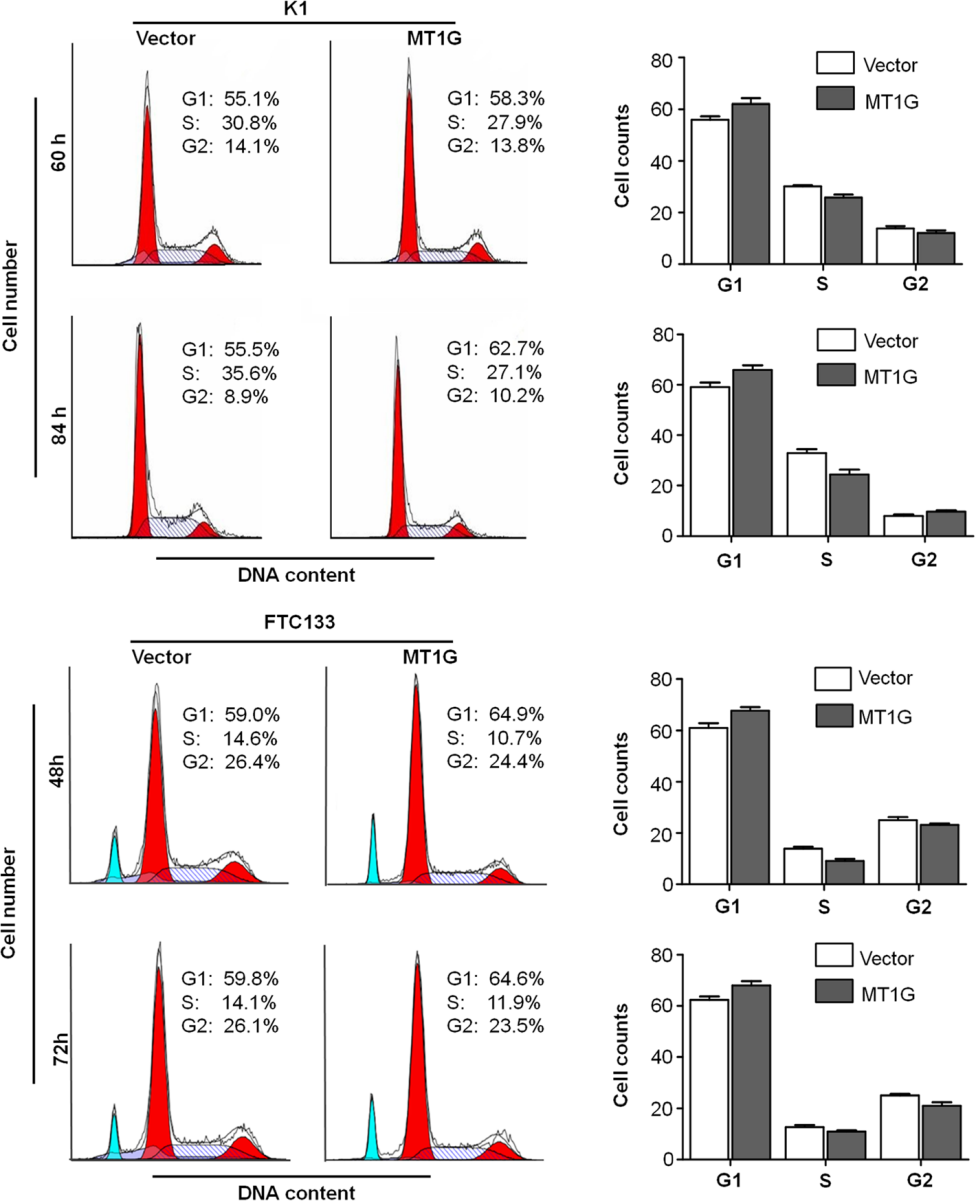
**MT1G induced thyroid cancer cell cycle arrest.** K1 and FTC133 cells transiently transfected with pEGFP-N1-*MT1G* or empty vector were cultured for the indicated times. DNA content was measured by flow cytometry to determine cell cycle fractions. Representative flow cytometric histograms of cells transfected with pEGFP-N1-*MT1G* and empty vector from three independent experiments were shown in left panel. The fraction of cells in each cell cycle phase was indicated in right panel.

**Figure 3:**
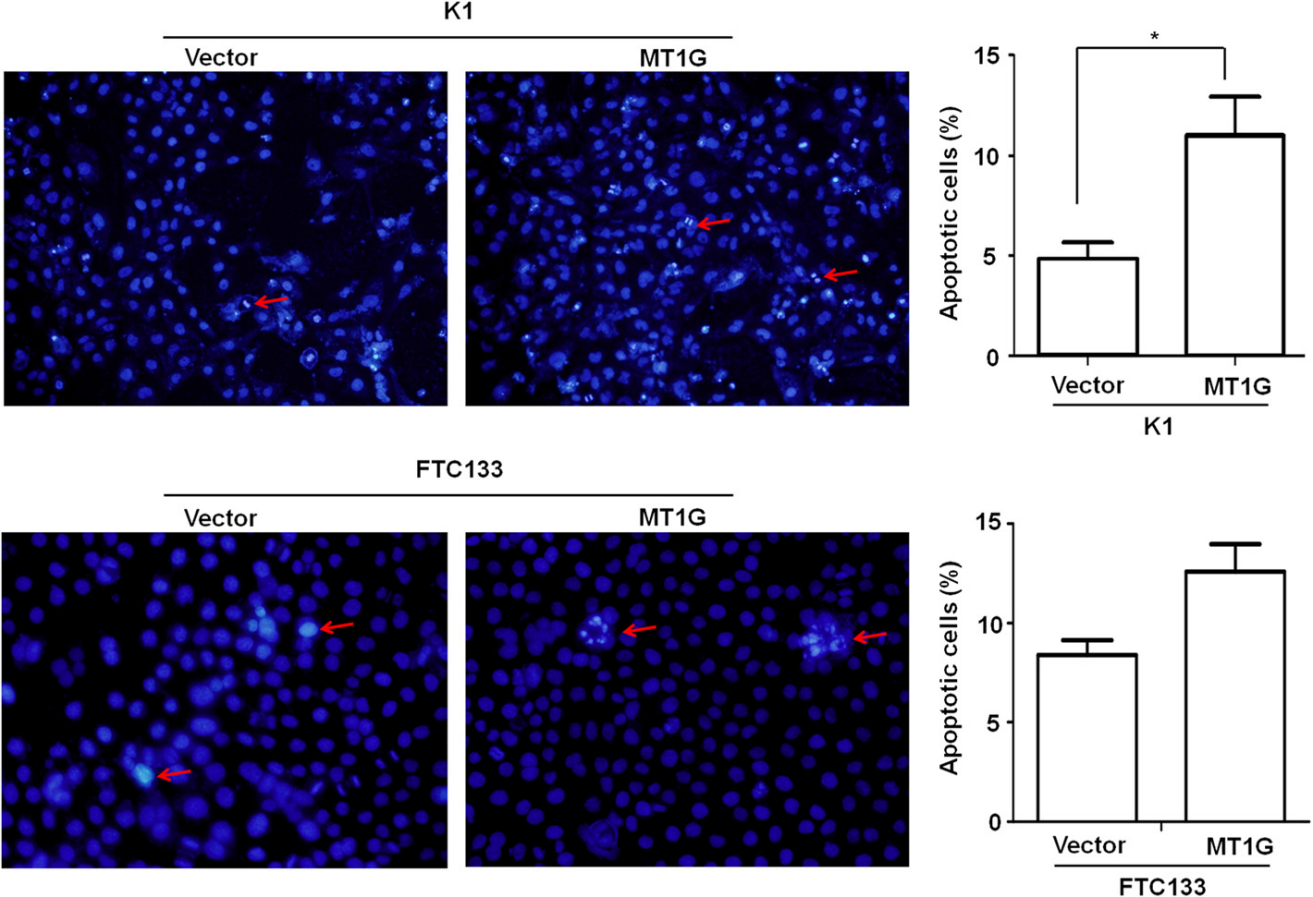
**MT1G induced thyroid cancer cell apoptosis.** K1 and FTC133 cells were transiently transfected with pEGFP-N1-*MT1G* or empty vector. After 48 h post-transfection, apoptotic cells were determined by Hoechst 33342 staining and observed under a fluorescent microscope. Selective apoptotic nuclei showing characteristics of apoptosis, including chromatin margination, condensation, and fragmentation, were indicated by arrows. Relative percentage of apoptotic cells in the *MT1G*-transfected and empty vector-transfected groups was presented in right panel. *, *P* <0.05.

Suppression of cell growth in cancer cells is usually associated with concomitant cell cycle arrest and activation of cell death pathways. We therefore examined the contribution of cell cycle arrest and apoptosis to the observed growth inhibition of *MT1G*-transfected cells. As shown in Figure 2, compared with empty vector, cell cycle was arrested at the G1 phase when cells were transfected with pEGFP-N1-*MT1G*. The percentage of G1 phase was increased from 55.9% to 62.1% at 60 h post-transfection, and from 59.1% to 65.9% at 84 h post-transfection in K1 cells, and from 61.0% to 67.7% at 48 h post-transfection, and from 62.4% to 68.0% at 72 h post-transfection in FTC133 cells, respectively. In addition, characteristic morphologies of apoptotic nuclei, such as chromatin condensation, margination and nuclear fragmentation, were more frequently observed in cells transfected with pEGFP-N1-*MT1G* compared with empty vector. As shown in Figure 3, the apoptotic cell number increased in *MT1G*-transfected cells compared with empty vector-transfected cells, particularly in K1 cells.

### MT1G inhibits thyroid cancer cell migration and invasion

**Figure 4:**
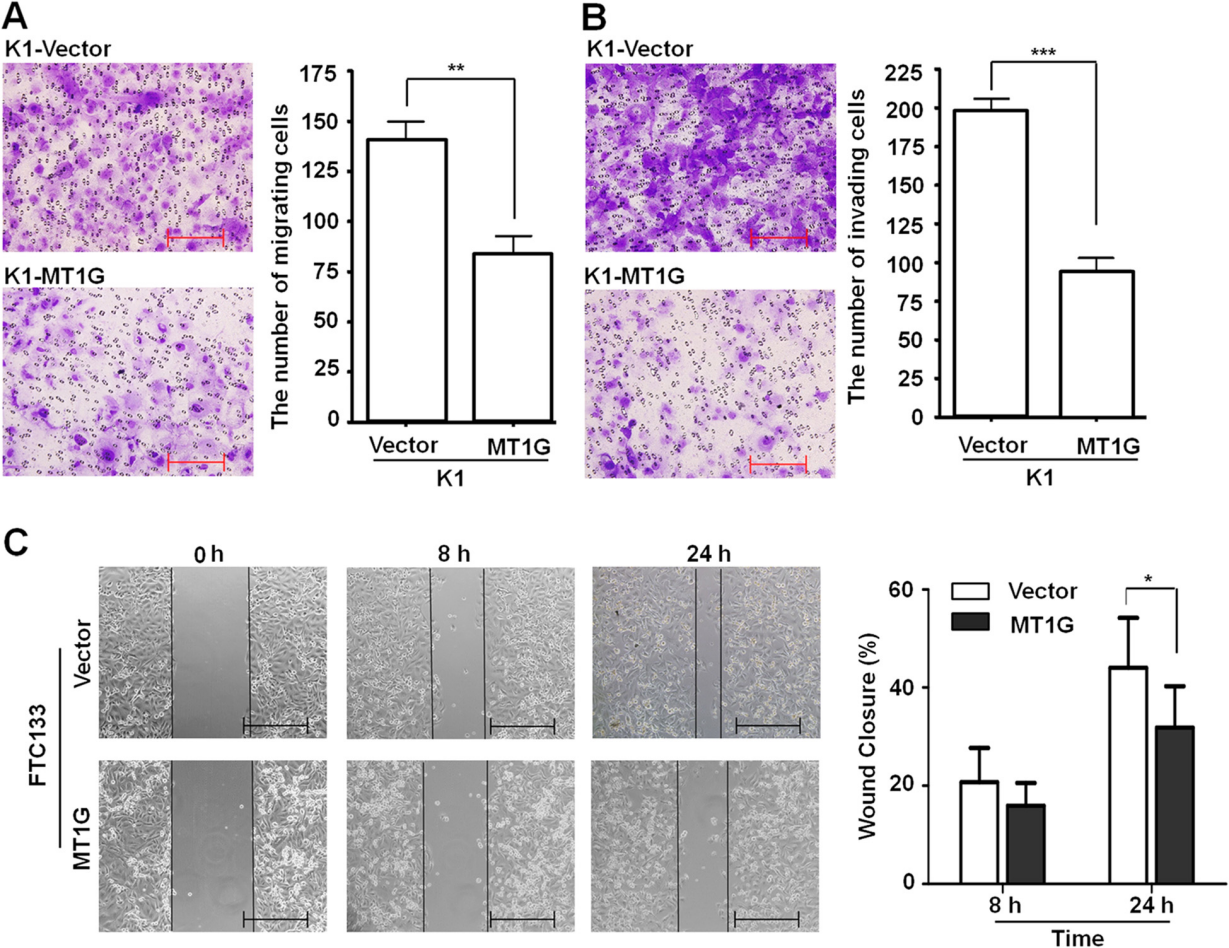
**MT1G inhibited the migration and invasion of thyroid cancer cells.** K1 cells stably transfected with pEGFP-N1-*MT1G* or empty vector were starved overnight and then seeded in the Transwell chambers without Matrigel for migration assay **(A)**, and coated with Matrigel for invasion assay **(B)**, respectively. Following a 24 h-culture, non-migrating (or non-invading) cells in the upper chamber were removed and migrating (or invading) cells were stained and calculated in four microscopic fields per sample. Shown are representative images of migrating (or invading) cells (left panels). The bar graphs (right panels), corresponding to left panels, show means ± SD of the numbers of migrating (or invading) cells from three independent experiments. **, *P* <0.01; ***, *P* <0.001. **(C)** FTC133 cells were transfected with pEGFP-N1-*MT1G* or empty vector. After 48 h of post-transfection, the scratch wound-healing assay was performed to evaluate the effect of MT1G on cell migration. Representative images of cell migration of FTC133 cells in scratch wound-healing assay were shown in left panel. The bar graphs show means ± SD. of gap width of wounds at the indicated times from three independent experiments (right panel). *, *P* <0.05.

In the present study, promoter methylation of *MT1G* was shown to increase the risk of lymph node metastasis in PTC patients. Thus, we next attempted to explore the effect of *MT1G* restoration on the migration and invasion of thyroid cancer cells. As shown in Figure 4A, for K1 cells, there was a significantly lower number of migrated cells in *MT1G*-transfected cells than empty vector-transfected cells (*P* <0.01), indicating that MT1G inhibited cancer cell migration. Furthermore, the Matrigel assays showed that the number of cells that passed through Matrigel-coated membrane into the lower chamber was significantly lower in *MT1G*-transfected K1 cells than empty vector-transfected K1 cells (*P* <0.001, Figure 4B). Cell migration and invasion assays were also performed in FTC133 cells using the same protocols. However, we failed to find any migrating or invading cells in both *MT1G*- and empty vector-transfected cells. Thus, scratch wound-healing assay was performed to evaluate cell migration in FTC133 cells. As shown in Figure 4C, the wound healing was markedly inhibited in *MT1G*-transfected cells as compared to empty vector-transfected cells. These observations suggest that MT1G inhibits the invasive potential of thyroid cancer cells.

### MT1G acts as a tumor suppressor via modulating the activity of PI3K/Akt pathway

**Figure 5:**
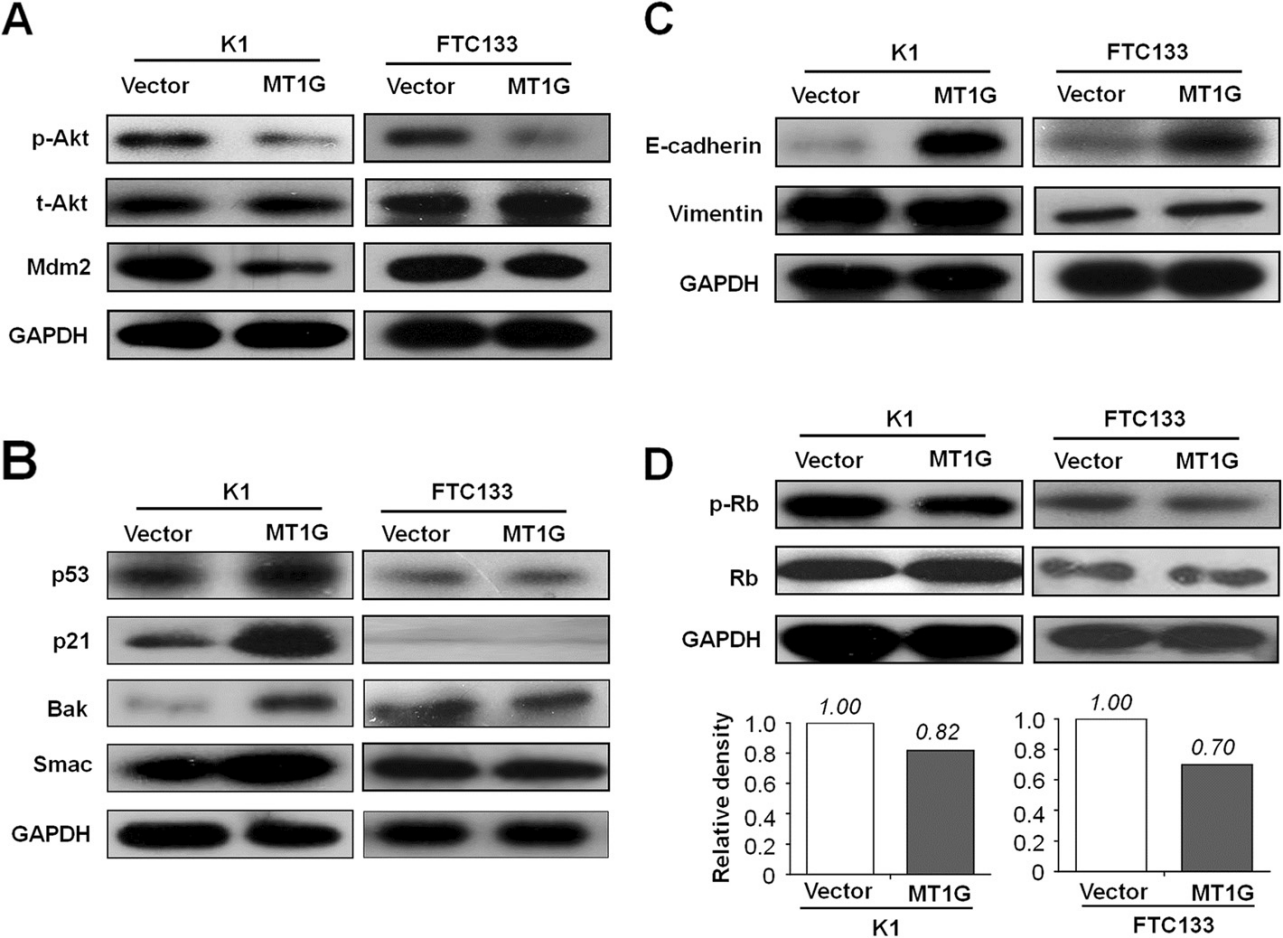
**MT1G inhibited the activity of PI3K/Akt pathway and phosphorylation of tumor suppressor Rb in thyroid cancer cells.** Thyroid cancer cell lines K1 and FTC133 stably transfected with pEGFP-N1-MT1G or empty vector were starved overnight and harvested. Cell lysates were collected and subjected to Western blotting assays. **(A)** The antibodies against phospho-Akt^Ser473^ (p-Akt), total-Akt (t-Akt), and Mdm2 were used to determine the effect of MT1G on the activity of PI3K/Akt signaling. **(B)** The effect of MT1G on the p53 signaling pathway was determined by blotting p53 and its downstream targets, p21, Bak and Smac. **(C)** The expression of E-cadherin and Vimentin were used to evaluate the effect of MT1G on cell migration and invasion. **(D)** The antibody against phospho-Rb^Ser811^ (p-Rb) was used to determine the effect of MT1G on the Rb/E2F signaling. Shown in the lower portion of the panel is a quantitative illustration of levels of p-Rb protein using densitometry to measure the density of the corresponding bands on Western blot shown in the upper portion of the panel. GAPDH was used for quality control of Western blotting.

**Figure 6:**
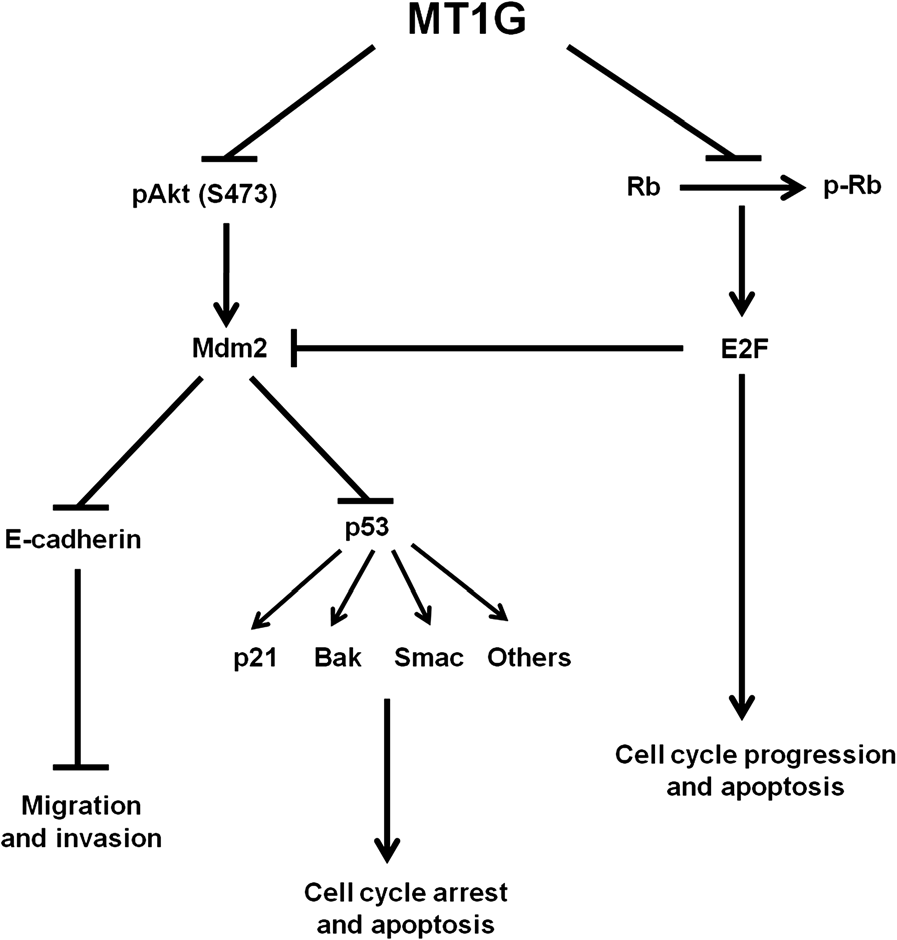
**Schematic diagram of *MT1G *as a tumor suppressor in thyroid cancer cells through regulating the PI3K/Akt and Rb/E2F pathways.***MT1G* restoration inhibited phosphorylation of Akt, leading to inhibition of cell growth. MT1G decreased Mdm2 expression through inhibiting the activity of Akt kinases. MT1G increased the stability of p53 and E-cadherin by the decreased ubiquitination of Mdm2, contributing to induction of cell cycle arrest and apoptosis and suppression of cell migration and invasion. MT1G also influenced cell growth, such as cell cycle progression and cell death, through inhibiting phosphorylation of Rb.

To gain insights into the downstream signaling pathways modulated by MT1G in tumor inhibition, we investigated the effect of MT1G on the activities of PI3K/Akt and MAPK pathways, which play a key role in cell proliferation and survival in human cancers, including thyroid cancer [5]. Our data showed that ectopic expression of *MT1G* inhibited phosphorylation of Akt in both K1 and FTC133 cells (Figure 5A). However, we did not find its effect on phosphorylation of Erk1/2 (data not shown). Next, we investigated the effect of MT1G on the expression of Mdm2, which can be regulated by the PI3K/Akt pathway [28-30]. As also shown in Figure 5A, we indeed observed that *MT1G* restoration decreased Mdm2 expression in thyroid cancer cells. It is well known that PI3K/Akt pathway can influence the activity and stability of tumor suppressor p53 through phosphorylation of Mdm2 [28-31]. Thus, we investigated the effect of MT1G on the p53 signaling pathways. As shown in Figure 5B, restoring *MT1G* expression increased the activity and stability of p53, and the expression of its downstream targets, including p21, Bak and Smac, in K1 cells. However, this phenomenon was not found in FTC133 cells because *TP53* gene is mutated in this cell line (see Additional file 1: Table S1), leading to p53 inactivation. These findings suggest that MT1G induces cell cycle arrest and apoptosis at least partially mediated by p53 signaling pathway. Collectively, MT1G inhibits thyroid cancer cell growth mainly through regulating PI3K/Akt signaling pathway (Figure 6).

To explore the molecular mechanism of MT1G contributing to thyroid cancer cell migration and invasion, we investigated the effect of MT1G on expression of E-cadherin and Vimentin, the altered expressions of which are hallmarks of epithelial-mesenchymal transition (EMT) allowing epithelial cells to separate from their neighbors and migrate to distant regions during tumor development [32]. As shown in Figure 5C, E-cadherin expression was dramatically up-regulated in the *MT1G*-transfected cells compared with empty vector-transfected cells. However, Vimentin expression was not significantly influenced by *MT1G* restoration. Additionally, we determined the mRNA expression of *E*-*cadherin*, *Vimentin*, and the transcription suppressors of *E*-*cadherin*, including *Snail*, *Slug*, and *Twist* in K1 and FTC133 cells. As shown in (see Additional file 1: Figure S3), the expression of these genes was not significantly different between MT1G-transfected cells and empty vector-transfected cells, suggesting that MT1G regulated E-cadherin expression at the post-transcriptional level. Taken together, our data suggest that MT1G inhibits cell migration and invasion by increasing the stability of E-cadherin (Figure 6).

Notably, we observed that MT1G slightly inhibited phosphorylation of tumor suppressor Rb, which plays a key role in regulating cell cycle and cell death [33,34], in the *MT1G*-transfected cells as compared to empty vector-transfected cells (Figure 5D), suggesting that MT1G might play a role in the control of cell proliferation partially through modulating the activity of Rb/E2F pathway (Figure 6).

## Discussion

In the present study, we found that *MT1G* expression was frequently absent or down-regulated in thyroid cancer cell lines, and was also significantly decreased in primary thyroid cancer tissues compared with non-malignant thyroid tissues, which was consistent with the previous studies [22,23]. These findings suggested that *MT1G* would be a candidate tumor suppressor in the pathogenesis of thyroid cancer. The reduced expression of *MT1G* is closely associated with promoter methylation, as confirmed by MSP assays and pharmacological DNA demethylation treatment in the present study and a previous study [23], implicating DNA methylation as a regulatory mechanism of *MT1G* inactivation in thyroid cancer. However, although there was a higher prevalence of *MT1G* hypermethylation in thyroid cancer tissues than in non-malignant thyroid tissues, the difference was not significant, which was consistent with a previous study in hepatocellular cancer [19]. Thus, we speculated that other epigenetic mechanisms such as histone modification, along with DNA methylation, may contribute to *MT1G* inactivation in thyroid carcinogenesis. In support of this, we treated thyroid cancer cells with a histone deacetylase inhibitor, SAHA, alone or in combination with 5-Aza-dC to explore the role of histone deacetylation in regulating *MT1G* expression. Our data showed that SAHA dramatically induced *MT1G* expression in thyroid cancer cells, suggesting that histone deacetylation may be another crucial mechanism of *MT1G* inactivation in thyroid cancer.

Down-regulation or silencing of *MT1G* might abolish tumor suppression so as to contribute to thyroid tumorigenesis. We thus tested the putative tumor suppressor function of *MT1G* in human thyroid cancer cells. *MT1G* restoration in thyroid cancer cells showed significant growth-suppressing effect by inhibiting cell proliferation and colony formation in the present study. In line with this finding, a previous study demonstrated that cell growth was inhibited in *MT1G*-reexpressed cells by both *in vitro* and *in vivo* assays [23]. Our data also showed that *MT1G* re-expression induced cell cycle arrest and apoptosis, further supporting its tumor suppressor function. Of note, *MT1G* hypermethylation significantly increased the risk of lymph node metastasis in PTC patients, as supported by our findings that *MT1G* restoration dramatically inhibited the migration and invasion of thyroid cancer cells.

Although the evidence has highlighted the importance of *MT1G* as an oncosuppressor in thyroid cancer, the precise molecular mechanisms remain largely unclear. To better understand the tumor suppressive effect of *MT1G* in thyroid tumorigenesis, we investigated the effect of *MT1G* on the activities of two major signaling pathways in thyroid cancer, including the PI3K/Akt and MAPK pathways. These two pathways are involved in propagation of signals from various cell membrane receptor tyrosine kinases into the nucleus, and regulate multiple cell processes, including cell proliferation, differentiation, and survival [5,35,36]. Our data showed that ectopic expression of *MT1G* strongly inhibited phosphorylation of Akt, but not Erk1/2, in thyroid cancer cells, suggesting that *MT1G* may play its tumor suppressor role through modulating the activity of PI3K/Akt pathway.

To explore the mechanism of *MT1G* contributing to induction of cell cycle arrest and apoptosis, we tested the effect of *MT1G* on p53 signaling pathways. Our findings showed that *MT1G* restoration increased the stability of p53 and the expression of its downstream targets, including p21, Bak, and Smac, in K1 cells, but not in FTC133 cells. Of the genes transcriptionally regulated by p53, p21^WAF/CIP1^ acts as a necessary mediator for the p53-mediated G1 arrest [37]. Bak, involving in p53-mediated mitochondrial apoptosis, is a pro-apoptotic Bcl-2 family protein which induces the release of apoptogenic factors, such as cytochrome *c* or Smac/DIABLO [38,39]. These data demonstrated that the effect of MT1G on cell cycle and cell death might be at least partially attributed to p53-mediated cell cycle arrest and apoptosis. With the consideration of decreased expression of Mdm2 induced by MT1G, the up-regulation of p53 is most likely caused by the reduced ubiquitination of Mdm2. Mdm2 functions as an E3 ubiquitin ligase, involving in eukaryotic protein degradation via ubiquitin proteasome system [40]. It decreases the stability of p53 by binding to its N-terminal transactivation domain (TAD), and therefore, stimulating its polyubiquinated degradation [41]. The previous studies provide strong evidences that active Akt binds to and phosphorylates Mdm2 at Ser166 and Ser186 to enhance protein stability. Furthermore, phosphorylated Mdm2 translocates more efficiently to the nucleus, where it can bind p53, resulting in enhanced p53 degradation [28-30]. This was supported by our findings that *MT1G* restoration inhibited phosphorylation of Akt and the expression of Mdm2, further contributing to increased stability of p53.

In the present study, we found that *MT1G* hypermethylation was an independent risk factor for lymph node metastasis in PTC. To be consistent with this, the previous studies showed the association of *MT1G* hypermethylation with poor prognosis in prostate cancer, hepatoblastoma and colorectal cancer [20,21,32]. Thus, we supposed that *MT1G* may play a role in the migration and invasion of thyroid cancer cells. Delightedly, our data showed that *MT1G* restoration increased E-cadherin expression, resulting in the inhibition of migration and invasion in thyroid cancer cells. Decreased expression of E-cadherin is a critical molecular event of epithelial-mesenchymal transition (EMT), which endows the epithelial cells with fibroblast-like properties and shows reduced intercellular adhesion and increased motility [32]. In oncogenic process, multiple signal transduction pathways may induce EMT. MAPK pathway, for example, has been shown to activate two transcription factors Snail and Slug, both of which are transcriptional repressors of E-cadherin [42,43]. Twist, another transcription factor, also induces loss of E-cadherin-mediated cell-cell adhesion and EMT [44]. However, our data showed that *MT1G* restoration did not affect the expression of these genes, suggesting MT1G-mediated E-cadherin up-regulation at a posttranscriptional level. A previous study revealed a novel role of Mdm2 in interaction with E-cadherin leading to its ubiquitination and degradation, which promotes cell motility and invasiveness [45], as supported by our findings that MT1G inhibited phosphorylation of Akt and the expression of Mdm2, ultimately contributing to increased stability of E-cadherin.

It is now clear that the Rb/E2F pathway is critical in regulating the initiation of DNA replication and plays a key role in controlling cell growth in human carcinogenesis [33]. We also found that *MT1G* re-expression slightly inhibited phosphorylation of Rb in the present study, implicating the effect of MT1G on cell growth at least partially through modulating the activity of Rb/E2F pathway. This finding was supported by a recent study that *SM22α* overexpression activated the Rb/E2F pathway through elevating *MT1G* expression in human hepatocarcinoma cells [46].

## Conclusions

In summary, our data showed that *MT1G* acted as a tumor suppressor, which was frequently inactivated by epigenetic alterations, such as promoter methylation and histone modification, in thyroid cancer. *MT1G* contributes to suppression of thyroid carcinogenesis by inhibiting cell growth and invasiveness, and inducing cell cycle arrest and apoptosis mainly through modulating the PI3K/Akt signaling pathway and partially through regulating the Rb/E2F pathway.

## Competing interests

The authors declare that they have no competing interests.

## Authors’ contributions

PH conceived and designed the experiments. JF, HJ, HG, and XM performed the experiments. NH and BS collected the patient materials. JF, MJ and PH analyzed the data. BS and PH contributed reagents/materials/analysis tools. JS and PH wrote the paper. All authors are in agreement with the content of the manuscript and this submission. All authors read and approved the final manuscript.

## Pre-publication history

The pre-publication history for this paper can be accessed here:

http://www.biomedcentral.com/1471-2407/13/462/prepub

## Supplementary Material

Additional file 1 Table S1 Characterization of genetic alteration in thyroid cancer cell lines used in this study. **Table S2.** RT-PCR primers used in this study. **Table S3.***MT1G* hypermethylation in thyroid cancer and goiter tissues. **Table S4.***MT1G* hypermethylation in PTC ― multivariable models assessing gender, age, tumor invasion, lymph node metastasis, tumor stage, and tumor recurrence (OR^1^ and 95% CI). **Figure S1.** MT1G down-regulation and frequent promoter hypermethylation in papillary thyroid cancer (PTC). **Figure S2.** MT1G inhibited thyroid cancer cell growth. **Figure S3.** The effect of MT1G on mRNA level of *E*-*cadherin*, *Vimentin*, *Snail*, *Slug*, and *Twist* in thyroid cancer cells. Click here for file
