# Supplementary material for: Metallothionein 1G functions as a tumor suppressor in thyroid cancer through modulating the PI3K/Akt signaling pathway
Source: BMC Cancer. 2013 Oct 8;13:462. doi: 10.1186/1471-2407-13-462 (PMC3851544; doi:10.1186/1471-2407-13-462)
Supplement: Additional file 1 Table S1 — Characterization of genetic alteration in thyroid cancer cell lines used in this study. Table S2. RT-PCR primers used in this study. Table S3.MT1G hypermethylation in thyroid cancer and goiter tissues. Table S4.MT1G hypermethylation in PTC ― multivariable models assessing gender, age, tumor invasion, lymph node metastasis, tumor stage, and tumor recurrence (OR1 and 95% CI). Figure S1. MT1G down-regulation and frequent promoter hypermethylation in papillary thyroid cancer (PTC). Figure S2. MT1G inhibited thyroid cancer cell growth. Figure S3. The effect of MT1G on mRNA level of E-cadherin, Vimentin, Snail, Slug, and Twist in thyroid cancer cells. [file 1471-2407-13-462-S1.doc]

**Additional file**

**Supplemental Tables**

**Table S1.** Characterization of genetic alteration in thyroid cancer cell lines used in this study

| **Cell lines** | **Origins** | **Genetic Alterations** | | | | | |
| --- | --- | --- | --- | --- | --- | --- | --- |
|  |  | RET HRAS PIK3CA BRAF TP53 others | | | | | |
| **K1** | PTC | WT | WT | E542K | V600E | R213R | -- |
| **BCPAP** | PTC | WT | WT | WT | V600E | D259Y | AKT1 copy gain |
| **IHH4** | PTC | WT | WT | WT | WT | WT | -- |
| **FTC133** | FTC | WT | WT | WT | WT | R273H | PTEN loss |
| **C643** | ATC | WT | G13R | WT | WT | R248Q | -- |
| **8305C** | ATC | WT | WT | WT | V600E | R273H | -- |

WT, wild type; --, No information

**Table S2**. RT-PCR primers used in this study

| Genebank  (Ref. No.) | Gene | Forward primer (5’-3’) | Reverse primer (5’-3’) | Product length (bp) | |
| --- | --- | --- | --- | --- | --- |
| NM_005950.1 | *MT1G* | GGAACTCTAGTCTCGCCTCG | GCATTTGCACTCTTTGCACT | | 105 |
| NM_004360.3 | *E-cadherin* | GACCGGTGCAATCTTCAAAA | CAGGTCTCCTCTTGGCTCTG | | 140 |
| NM_003380.3 | *Vimentin* | CTGGATTTCCTCTTCGTGGA | CGAAAACACCCTGCAATCTT | | 133 |
| NM_005985.3 | *Snail* | TCCAGAGTTTACCTTCCAGCA | CTTTCCCACTGTCCTCATCTG | | 218 |
| NM_003068.4 | *Slug* | CTACAGCGAACTGGACACACA | GCCCCAAAGATGAGGAGTATC | | 200 |
| NM_000474.3 | *Twist* | GTCCGCAGTCTTACGAGGAG | GTCTGAATCTTGCTCAGCTTGTC | | 148 |
| NR_003286.2 | *18S* | CGCCGCTAGAGGTGAAATTC | CTTTCGCTCTGGTCCGTCTT | | 52 |
| NM_001101.3 | *β-Actin* | GCACAGAGCCTCGCCTT | GTTGTCGACGACGAGCG | | 93 |

**Table S3. *MT1G* hypermethylation in thyroid cancer and goiter tissues**

| Gene | PTC (n=178) | FTC (n=16) | MTC (n=9) | ATC (n=9) | Total (n=212) |  | Goiter  (n=32) |
| --- | --- | --- | --- | --- | --- | --- | --- |
|  | N (%) | N (%) | N (%) | N (%) | N (%) |  | N (%) |
| *MT1G* | 56 (31.5%) | 4 (25.0%) | 2 (22.2%) | 2 (22.2%) | 64 (30.2%) |  | 6 (18.8%) |

**Table S4**. *MT1G* hypermethylation in PTC ― multivariable models assessing gender, age, tumor invasion, lymph node metastasis, tumor stage, and tumor recurrence (OR^1^ and 95% CI)

| **Variable** | **OR^1^** | **95%CI** | ***P* value** |
| --- | --- | --- | --- |
| Gender | 0.89 | 0.42-1.89 | 0.77 |
| Age^2^ | 1.01 | 0.59-1.70 | 0.99 |
| Tumor invasion | 0.58 | 0.28-1.21 | 0.15 |
| Lymph node metastasis | 2.40 | 1.19-4.83 | 0.01* |
| Tumor stage^3^ | 1.14 | 0.67-1.93 | 0.63 |
| Tumor recurrence | 0.88 | 0.33-2.36 | 0.81 |

1 OR: odds ratio with 95% confidence interval; 2 Age (≤30y; 30-50y; 50-70y; >70y); 3 Tumor stage (I, II, III, IV); * Significant at *P* <0.05.

**Figure legends**

**Figure S1.** **MT1G down-regulation and frequent promoter hypermethylation in papillary thyroid cancer (PTC). (A)** MT1G mRNA expression was significantly down-regulated in primary PTC tissues compared with non-malignant thyroid tissues as determined by real-time quantitative RT-PCR. MT1G expression level was normalized with 18S mRNA level. Data are presented as mean ± SD. *** , *P* <0.001. (**B**) Schematic representation of the 5’ regions of MT1G. A typical CpG island spans the promoter region of MT1G. Each vertical bar represents a single CpG site. The region of exon 1 is shown. The transcription start site is indicated by a curved arrow, and the position of MSP primers was indicated by short arrows. (**C**) Promoter methylation of MT1G in primary PTC samples was determined by methylation-specific PCR (MSP). In vitro methylated DNA was used as positive control for methylated gene (P), bisulfite-modified normal leukocyte DNA as positive control for unmethylated gene (N), and H2O as blank control to confirm the specificity of MSP. Details are described in the Materials and Methods. Mk, DNA marker; M, methylated gene; U, unmethylated gene. PTC-1 and -2 present two PTC samples with different methylation status of MT1G.

**Figure S2. MT1G inhibited thyroid cancer cell growth.** (**A**) Ectopic expression of *MT1G* mRNA in K1 (upper lane) and FTC133 (lower lane) cells was evidenced by semi-quantitative and real-time quantitative RT-PCR. *β-actin* was used as the quality control for semi-quantitative RT-PCR analysis. *18S* mRNA was used as a normalized control for real-time quantitative RT-PCR. *** , P <0.001. (**B**) MT1G inhibited cell proliferation in K1, FTC133, C643 and BCPAP cells. *, *P* <0.05; **, *P* <0.01; ***, *P* <0.01. (**C**) MT1G inhibited cell growth analyzed by colony formation assay. Upper panel shows the representative images of colony formation in K1 and FTC133 cells transfected with pEGFP-N1-MT1G or empty vector. Quantitative analysis of colony numbers is shown in the lower panel. Details are described in the Materials and Methods. Data are presented as mean ± SD of values from three different assays. *, *P* <0.05.

**Figure S3. The effect of MT1G on mRNA level of *E-cadherin*, *Vimentin*, *Snail*, *Slug*, and *Twist* in thyroid cancer cells.** Total RNA was extracted from K1 and FTC133 cells stably transfected with pEGFP-N1-MT1G or empty vector. Real-time quantitative RT-PCR was then preformed to analyze the expression of *E-cadherin*, *Vimentin*, *Snail*, *Slug*, and *Twist*. *MT1G* expression level was normalized with *18S* mRNA level. Data are presented as mean ± SD.


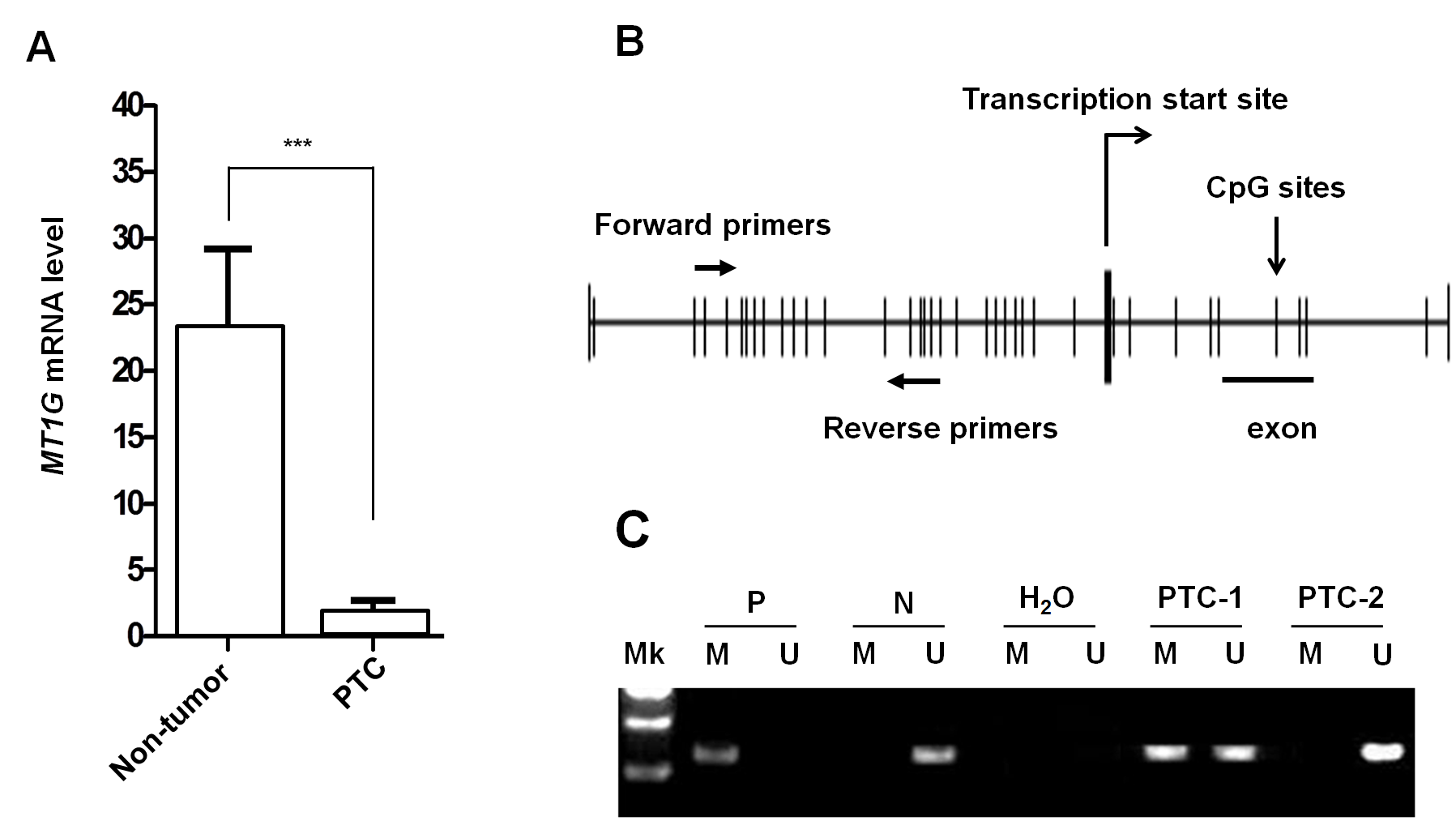


**Supplemental Figure S1**

**
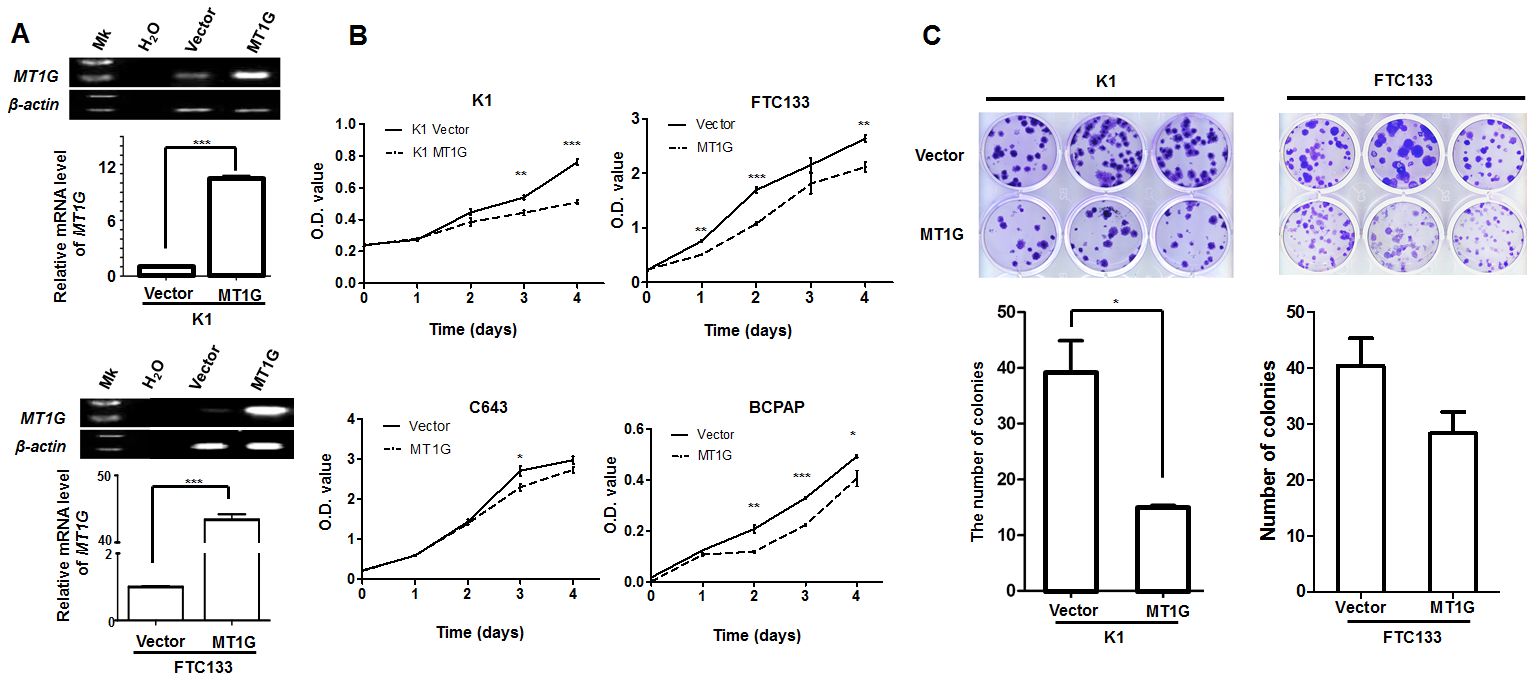
**

**Supplemental Figure S2**

**
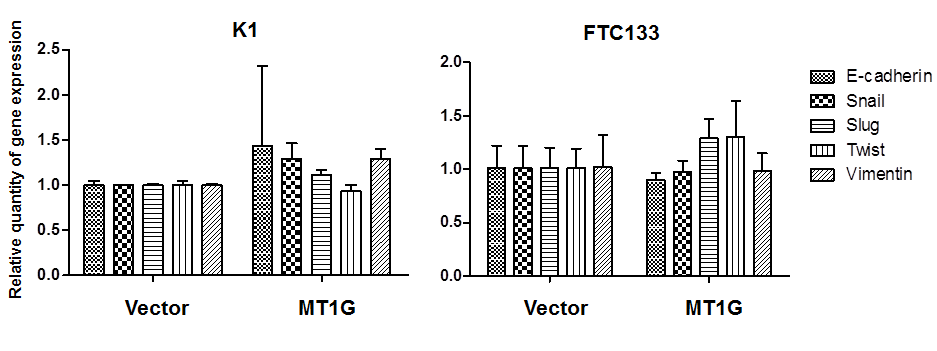
**

**Supplemental Figure S3**
